# Automotive Health 2.0: Steering Toward Proactive Preventive Care

**DOI:** 10.1016/j.mcpdig.2025.100334

**Published:** 2025-12-26

**Authors:** Dominik Naumann, Tatjana Amler, Doreen Schoeppenthau, Sergej Holzmann, Jörg Preißinger, Matthias Franz, Heyo K. Kroemer, Alexander Meyer

**Affiliations:** a Deutsches Herzzentrum der Charité, Institute for Artificial Intelligence in Medicine, Berlin, Germany; b Department of Cardiothoracic and Vascular Surgery, Deutsches Herzzentrum der Charité, Berlin, Germany; c DZHK (German Centre for Cardiovascular Research), Partner Site Berlin, Germany; d Charité – Universitätsmedizin Berlin, Corporate Member of Freie Universität Berlin and Humboldt-Universität zu Berlin, Berlin, Germany; e BMW Group Research and Technology, Munich, Germany; f Department of Cardiology, Angiology and Intensive Care Medicine, Deutsches Herzzentrum der Charité, Berlin, Germany; g Berlin Institute for the Foundations of Learning and Data—TU Berlin, Berlin, Germany

## Abstract

Cardiovascular and chronic disease prevention remains limited by episodic, clinic-based assessments that fail to capture physiological changes arising in daily life. As mobility constitutes one of the most stable and repetitive environments people inhabit, vehicles offer a unique setting for subliminal, continuous health monitoring. This narrative presents the rationale and foundational framework for Automotive Health 2.0, a clinically oriented paradigm that transforms connected vehicles into validated platforms for physiological sensing, data integration, and proactive care delivery. Building on existing in-cabin cameras, radar, and microphones, multimodal algorithms enable unobtrusive estimation of cardiovascular, respiratory, and behavioral parameters during routine driving. Technological innovation lies in combining these signals with artificial intelligence-driven analytics to detect early disease signatures, support dynamic risk assessment, and enable adaptive telemonitoring directly linked to electronic health records. Clinically, this approach distinguishes regulatory-grade monitoring from consumer wellness tools by prioritizing accuracy, reproducibility, and integration with established workflows. Patients gain earlier detection and more equitable access to preventive care; clinicians receive continuous actionable data, and health systems benefit from scalable population-level monitoring. Automotive Health 2.0 positions the vehicle as a novel extension of the health care ecosystem, embedding validated prevention seamlessly into everyday life.

Preventive medicine will continue to fall short unless it is woven into daily life, capturing health data continuously and unobtrusively without adding tasks for the individual. Although digital health tools have proliferated, real-world impact remains modest, as most provide limited ability to build individualized risk trajectories and require users to remember, wear, charge, or operate them — behaviors that fade quickly. Even when data are collected, they are often confined to proprietary applications, disconnected from electronic health records and clinical workflows. For prevention to become truly cost-effective, it must align with principles of predictive, preventive, and personalized medicine, enabling continuous phenotyping, dynamic risk stratification, and tailored interventions in both primary and secondary care.^1^ The connected vehicle, already equipped with advanced sensors, edge computing, and continuous connectivity, offers a uniquely stable environment for such individualized monitoring. People spend hundreds of hours annually in cars, creating a consistent physiological baseline from which deviations can be detected. Previous in-vehicle health initiatives have been fragmented, limited to single variables and clinically disconnected.^2^ Automotive Health 2.0, as outlined in the Figure below, proposes an integrated framework in which multimodal cardiovascular, respiratory, behavioral, and environmental data are fused using artificial intelligence to build longitudinal, person-specific profiles. These profiles support early disease detection, individualized risk trajectories, and preventive measures tailored to patient phenotype and clinical context.

**Figure:**
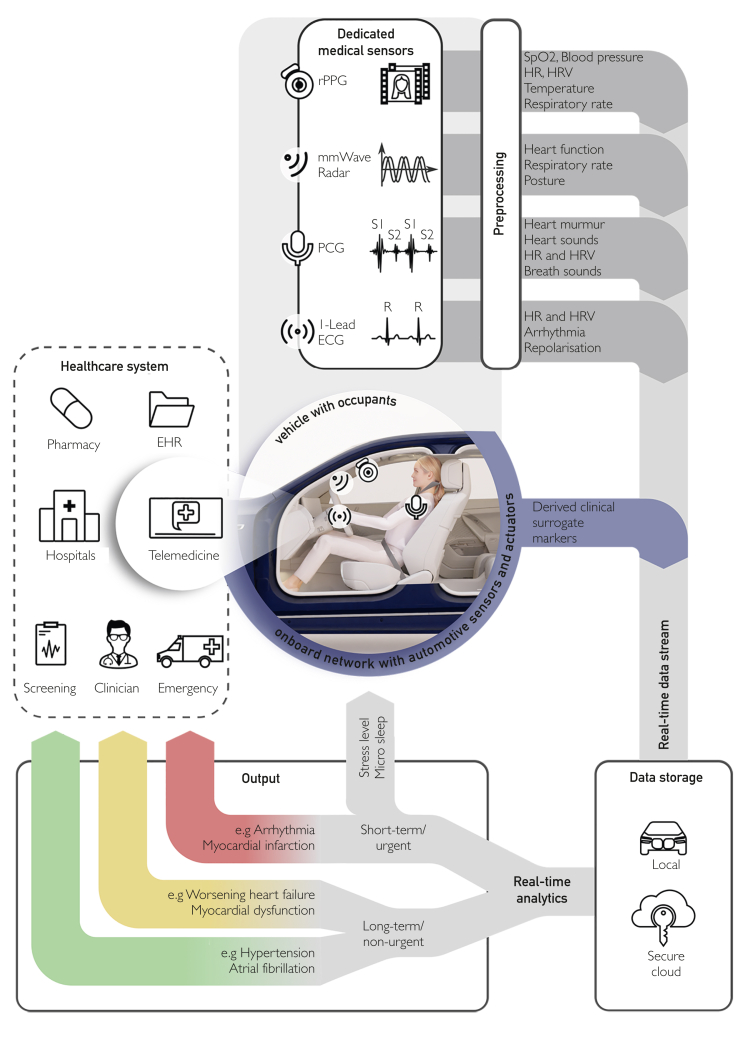
Automotive Health 2.0: AI-enabled, multimodal in-vehicle health monitoring and clinical integration. This conceptual framework illustrates how connected vehicles could evolve into intelligent platforms for continuous health monitoring and proactive clinical decision support. At its core is a multimodal in-cabin sensor array enabling subliminal, longitudinal assessment of physiological and behavioral parameters. Existing technologies, including cabin-facing cameras, radar, microphones, and environmental detectors can be repurposed or supplemented with medical-grade sensors. These can estimate heart rate (HR), heart rate variability (HRV), and heart rhythm (via single-lead electrocardiogram [ECG], radar, or phonocardiography [PCG]), blood pressure (via pulse transit time), respiratory rate, oxygen saturation (SpO_2_), and body temperature using contactless methods such as remote photoplethysmography (rPPG) or voice analysis. Additional metrics include stress level, microsleep detection, and driving behavior patterns as surrogate markers of functional and autonomic status. Onboard edge computing processes signals in real time, performing noise filtering, segmentation, and quality assessment. Data are triaged by clinical urgency: life-threatening events such as malignant arrhythmias or loss of consciousness trigger immediate alerts to emergency medical services (EMS) and, if required, autonomous vehicle control to bring the car to a safe stop. Indicators of chronic or subacute conditions — such as arterial hypertension, atrial fibrillation, or early heart failure decompensation — are forwarded to the treating physician and stored for longitudinal tracking. When clinically indicated, data are transmitted securely to cloud infrastructures for advanced analytics and integrated into electronic health records (EHRs) or clinical decision support systems. By combining physiological, behavioral, and environmental data, Automotive Health 2.0 aims to enable early disease detection, dynamic risk stratification, and personalized, adaptive in-cabin interventions — embedding preventive care seamlessly into daily life. AI, artificial intelligence.

The rationale is compelling, as noncommunicable diseases remain the leading global cause of mortality, largely driven by risk factors detected too late.^3^ Many at-risk individuals attend medical evaluations only when disease is advanced, while health systems remain focused on treatment rather than prevention. The WHO’s 2023-2030 Digital Health Strategy for Europe calls for embedding technologies into daily environments — an aim achievable by repurposing vehicles as sensor-rich spaces.^4^ Existing safety and comfort systems including cabin-facing cameras, radar, microphones, and environmental detectors can be repurposed for medical-grade monitoring, enabling continuous data collection without additional devices.^5^

Artificial intelligence is the catalyst that transforms in-vehicle data into actionable, personalized insights. Deep learning models can fuse physiological, behavioral, and contextual signals, enabling real-time risk assessment and longitudinal profiling in a familiar environment. Transformer-based algorithms, already shown to improve cardiovascular risk prediction, could similarly process in-vehicle data to identify subtle physiological changes.^6^ Continuous monitoring enables early detection of arterial hypertension and atrial fibrillation or signs of early heart failure deterioration.^7^ Integration with telemedicine platforms linked to electronic health records could enable continuous monitoring and support proactive, phenotype-tailored escalation of care.^8^ In acute events such as malignant arrhythmias or seizures, systems could trigger emergency medical services and, if necessary, initiate autonomous vehicle control to safeguard passengers and mitigate crash risk.

The potential, however, extends beyond cardiovascular disease. Cabin microphones and remote photoplethysmography may enable detection of abnormal breathing patterns, cough, or oxygen desaturation, supporting early diagnosis and telemonitoring of asthma and chronic obstructive pulmonary disease.^9^ Voice biomarkers, facial expression analysis, heart rate variability, and driving behavior may provide markers of stress, anxiety, or depression.^10^ Driving performance, reaction times, and decision-making patterns could serve as digital biomarkers of cognitive decline. Collectively, these applications indicate that Automotive Health 2.0 could become a platform for multidimensional preventive medicine. Federated and continual learning allow these models to evolve with each user, refining predictions without compromising data privacy and supporting population-level risk assessment while preserving individualized precision.^11^

At a population level, the potential is substantial. In rural and underserved communities, where access to regular screening remains limited, automotive health monitoring could embed preventive care into routine mobility. Extending monitoring from drivers to all occupants could enable applications in ridesharing, taxi fleets, and public transport, multiplying preventive reach and equity impact. Automotive Health 2.0 also holds meaningful benefits across stakeholders. Patients gain personalized preventive interventions, clinicians receive continuous, context-rich data that enhance decision-making, and health systems gain a scalable platform that supports earlier intervention, reduces avoidable hospitalizations, and improves population-level preventive care.

Real-world implementation requires rigorous clinical validation, technical robustness under variable environmental and motion conditions, and reproducibility across patient groups to ensure reliable individualized monitoring. Human factors are equally important: acceptance, trust, and potential psychological burden must be assessed to avoid surveillance-related anxiety. Clear frameworks for consent and data governance as well as ownership and safety are prerequisites for public trust. Regulatory pathways must define clinical utility, safety, and liability. Evidence standards should match those of medical devices with transparent reporting of accuracy, failure modes, and clinical outcomes to ensure that in-vehicle monitoring delivers meaningful improvements. Above all, the focus must remain on generating clinically actionable, personalized insights rather than consumer-grade approximations. Demonstrating value for both users and health systems through prospective studies and real-world evidence will be crucial for adoption and scalability.

Automotive Health 2.0 is not intended to replace conventional screening but to act as a personalized safety net, embedding preventive monitoring into the flow of daily life. By aligning advances in connected vehicles, sensor technologies, and artificial intelligence with clinical validation, telemedical integration, and ethical safeguards, vehicles could evolve into a distributed infrastructure for predictive, preventive, and personalized health care, transforming commuting into an extension of care.

## Potential Competing Interests

All authors are currently involved in an academic-industry cooperation between Charité—Universitätsmedizin Berlin and BMW Group Research and Technology. The authors report no other competing interests.
